# Autonomic Modulation in Patients with Heart Failure Increases Beat-to-Beat Variability of Ventricular Action Potential Duration

**DOI:** 10.3389/fphys.2017.00328

**Published:** 2017-05-29

**Authors:** Bradley Porter, Martin J. Bishop, Simon Claridge, Jonathan Behar, Benjamin J. Sieniewicz, Jessica Webb, Justin Gould, Mark O'Neill, Christopher A. Rinaldi, Reza Razavi, Jaswinder S. Gill, Peter Taggart

**Affiliations:** ^1^Department of Imaging Sciences and Biomedical Engineering, Kings College LondonLondon, United Kingdom; ^2^Cardiology Department, Guy's and St. Thomas' HospitalLondon, United Kingdom; ^3^Department of Cardiovascular Sciences, University College LondonLondon, United Kingdom

**Keywords:** arrhythmia, action potential duration, sympathetic nervous system, activation recovery interval, beta-blocker, heart failure

## Abstract

**Background:** Exaggerated beat-to-beat variability of ventricular action potential duration (APD) is linked to arrhythmogenesis. Sympathetic stimulation has been shown to increase QT interval variability, but its effect on ventricular APD in humans has not been determined.

**Methods and Results:** Eleven heart failure patients with implanted bi-ventricular pacing devices had activation–recovery intervals (ARI, surrogate for APD) recorded from LV epicardial electrodes under constant RV pacing. Sympathetic activity was increased using a standard autonomic challenge (Valsalva) and baroreceptor indices were applied to determine changes in sympathetic stimulation. Two Valsalvas were performed for each study and were repeated, both off and on bisoprolol. In addition sympathetic nerve activity (SNA) was measured from skin electrodes on the thorax using a novel validated method. Autonomic modulation significantly increased mean short-term variability in ARI; off bisoprolol mean STV increased from 3.73 ± 1.3 to 5.27 ± 1.04 ms (*p* = 0.01), on bisoprolol mean STV of ARI increased from 4.15 ± 1.14 to 4.62 ± 1 ms (*p* = 0.14). Adrenergic indices of the Valsalva demonstrated significantly reduced beta-adrenergic function when on bisoprolol (Δ pressure recovery time, *p* = 0.04; Δ systolic overshoot in Phase IV, *p* = 0.05). Corresponding increases in SNA from rest both off (1.4 uV, *p* < 0.01) and on (0.7 uV, *p* < 0.01) bisoprolol were also seen.

**Conclusions:** Beat-to-beat variability of ventricular APD increases during brief periods of increased sympathetic activity in patients with heart failure. Bisoprolol reduces, but does not eliminate, these effects. This may be important in the genesis of ventricular arrhythmias in heart failure patients.

## Introduction

Exaggerated beat-to-beat variability of repolarization (BVR) is strongly associated with pro-arrhythmia and is modulated by sympathetic activity (Shen and Zipes, 2014; Baumert et al., 2016). BVR is an intrinsic property of cardiac myocytes and can be observed at all levels from the ventricular action potential in the single cardiac cell to the QT interval in the ECG (Zaniboni et al., 2000; Tereshchenko et al., 2010; Heijman et al., 2013; Baumert et al., 2016). A growing body of experimental and computational work is providing insight into the potential mechanisms underlying BVR and the modulatory role of sympathetic stimulation at the level of the ventricular action potential duration (APD) (Heijman et al., 2013; Johnson et al., 2013; Pueyo et al., 2016). However, information on ventricular APD behavior in humans at present relies largely on QT interval measurements from the body surface ECG (Piccirillo et al., 2001, 2006; Malik, 2008; Porta et al., 2011; Baumert et al., 2016). While QT measurements have provided a great deal of valuable information, direct extrapolation to APD is not possible from these global recordings. Furthermore, such measurements are usually made with uncontrolled cycle length which complicates interpretation in view of the strong cycle length dependence of APD (Boyett and Jewell, 1978). Measurements of BVR from direct recordings of APD with controlled cycle length in patients have, to the best of our knowledge, not yet been obtained.

We have previously reported the measurement of local ventricular APD in patients with heart failure undergoing resynchronization therapy with bi-ventricular pacing (Chen et al., 2013). The implanted biventricular pacing devices (CRT) enable recordings to be made of local unipolar electrograms from the left ventricular epicardial lead while simultaneously pacing from the right ventricular lead in order to maintain constant cycle length. From the local electrograms, activation recovery intervals (ARI) may be derived as a conventional surrogate measure of APD (Wyatt et al., 1981; Coronel et al., 2006; Potse et al., 2009). We here report studies in patients with heart failure of beat-to-beat variability of ventricular APD (BBV-APD) during constant cycle length following an autonomic challenge. Studies were performed both with and without beta-adrenergic blockade (bisoprolol).

## Methods

### Ethical approval

The study was approved by the West London Ethics Committee and conformed to the standards set by the Declaration of Helsinki (latest revision: 64th WMA General Assembly). Informed consent was obtained in writing from all subjects.

### Subjects

Studies were performed in 11 ambulatory heart failure patients (all male, age 58–76) who were recipients of a CRT device (Quadra Assura MP™ CRT-D, St. Jude Medical). Patient characteristics are shown in Table 1.

**Table 1:** **Patient characteristics**.

**Subject**	**Age (years)**	**Gender**	**Etiology**	**NYHA Class**	**Ejection fraction (%)**	**Bisoprolol dose**
1	75	M	ICM	1	34	10 mg
2	75	M	ICM	2	33	10 mg
3	58	M	NICM	2	44	Nil
4	64	M	ICM	3	19	3.75 mg
5	61	M	ICM	2	22	7.5 mg
6	64	M	NICM	2	41	5 mg
7	69	M	ICM	2	40	5 mg
8	65	M	NICM	3	35	1.25 mg
9	76	M	NICM	1	63	Nil
10	71	M	NICM	2	42	7.5 mg
11	70	M	ICM	2	15	Nil
Range	58–76			1–3	15–63	
Mean ± *SD*	68 ± 6				35 ± 13	

*ICM, Ischaemic cardiomyopathy; NICM, Non-ischaemic cardiomyopathy*.

### Physiological recordings

**Figure 1:**
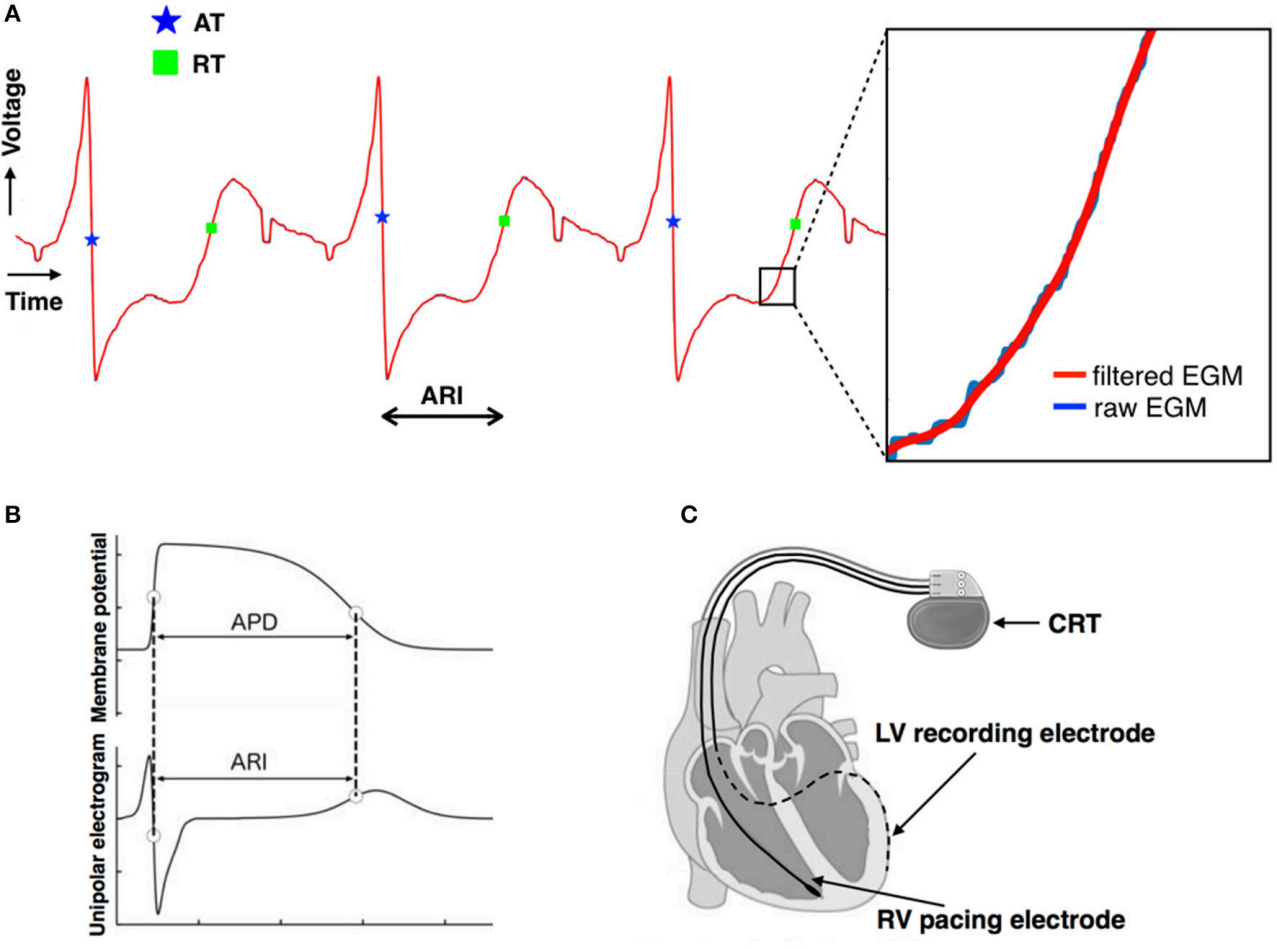
**(A)** Unipolar electrograms recorded from the left ventricular lead demonstrating the good quality of the signals. The moment of local activation (blue star) and of local repolarization (green square) are shown. The lack of any significant distortion of the signal by filtering is shown in the insert. **(B)** Relationship between the unipolar electrogram and the intracellular ventricular transmembrane potential showing correspondence between activation recovery interval (ARI) and action potential duration (APD). **(C)** Biventricular pacing device (CRT) programmed to pace from the right ventricular lead (solid line) and record a local electrogram from the left ventricular lead (dashed line).

Heart rate was fixed by constant right ventricular pacing using their implanted CRT device. The rate was chosen as the minimum rate required to maintain continuous capture at a fixed cycle length. A minimum adaptation period of 10 min took place prior to any recordings. The cycle length remained constant throughout the entire study and the same cycle length was chosen when studies were repeated on beta-blockade. The left ventricular epicardial lead was used to record unipolar electrograms, sampled at 512 Hz (Figure 1). The pacing programmer used to alter the pacing settings in this study allowed storage of five separate recordings of 30 s duration.

Arterial blood pressure was measured non-invasively using a finger cuff (Finometer pro, Finapres Medical Systems B.V., Amsterdam, The Netherlands). The signals were digitized by the MP150 System using AcqKnowledge software (Goleta, CA) and sampled at 1 kHz.

In addition sympathetic nerve activity (SNA) recordings were made using a novel method from a pair of bipolar skin electrodes in the Lead II position as in Doytchinova et al. (2017). The signals were digitized by the MP150 System using AcqKnowledge software (Goleta, CA) and sampled at 10 kHz.

### Protocol 1

Recordings were made with the subject seated upright. Physiological recordings were made simultaneously using multiple recording channels. Beta-adrenergic blocking agents (bisoprolol) were discontinued for 5 days prior to the study, which was sufficient wash-out time for all drugs in this population (see Table 1 for patient-specific dosage).

Subjects were asked to perform the Valsalva maneuver (forced expiration against a fixed resistance; Doytchinova et al., 2017) for 10 s. Physiological recordings were made continuously before, during and after the procedure. Following a 10 min recovery period a second Valsalva maneuver was performed and recordings were made as per the first.

### Protocol 2

Eight subjects returned to repeat the same protocol as above but without discontinuing their beta-blockers (Bisoprolol in all 8). Three patients could not be included as beta-blockers were not part of their regular medication.

### Analysis of data

#### ARI analysis

**Figure 2:**
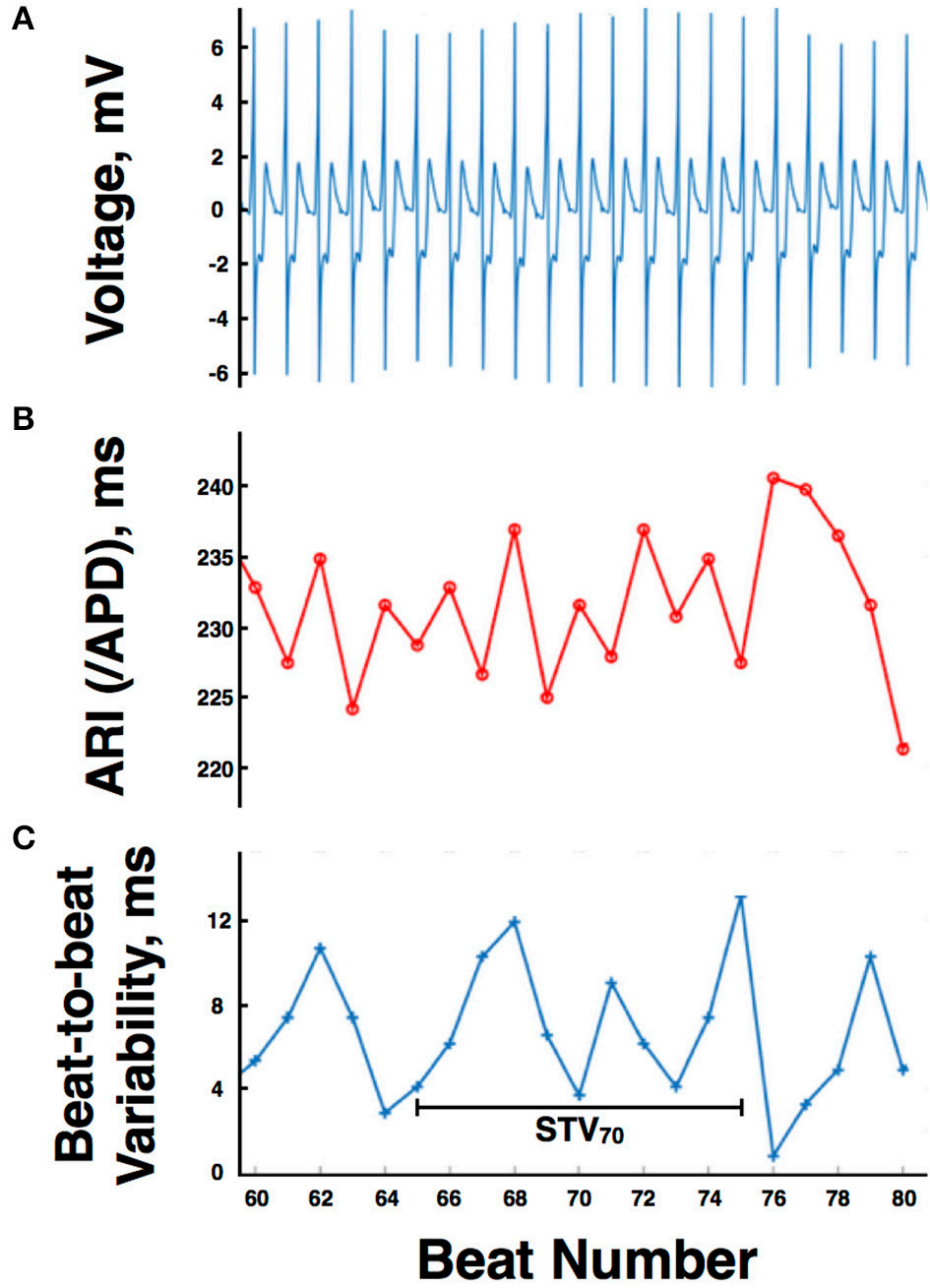
**(A)** Example of the digitized electrogram trace during a 20 s period of rest. **(B)** Computed activation recovery interval (ARI) values for the same electrogram trace. **(C)** Magnitude of the beat-to-beat variation in ARIs during the example 20 s period of rest. APD, action potential duration, STV, short-term variability.

Each 30 s output from the CRT device was digitized and truncated to form one long data sequence of ~150 s in length. Any possible overlap between successive 30 s sequences was found by searching for matching traces at the start/end of successive traces and was removed. Raw, digitized electrogram (EGM) traces were then lowpass filtered at 80 Hz which removed high-frequency noise, but maintained the sharp activation gradients required to identify activation times. Figure 1A shows the fidelity of the EGM traces, showing both the raw trace (blue) and the filtered trace (red). Figure 2A shows an example of the digitized EGM trace during a 20 s period of rest showing multiple beats.

Activation times (ATs) were defined as the point of maximum negative downslope of the unipolar electrogram following the R-wave. Repolarization time (RT) was defined using the Wyatt method as the point of maximum upslope of the T-wave (Wyatt et al., 1981; Coronel et al., 2006; Potse et al., 2009; Hanson et al., 2014). ARIs for each (*i*th) beat were then computed as.

$$\begin{array}{l} ARI_{i}=RT_{i}-AT_{i} \end{array}$$

Figure 1A shows the identification of the AT (blue star) and RT (green square) for a single ARI. Figure 2B shows the computed ARI values for the corresponding EGM trace in Figure 2A. The magnitude of the mean beat-to-beat changes in ARI during resting recordings was inline with previous studies (Hanson et al., 2014). Figure 2C shows the corresponding magnitude of the beat-to-beat variation in ARIs during the example 20 s period of rest.

To quantify the variation in ARIs between beats and examine how this may change during sympathetic stimulation, short-term variability (STV) of ARIs was computed, as previously defined (Johnson et al., 2013). Specifically, a 10-beat moving window was defined throughout the recordings and STV at the beat *i*th computed as.

$$STV_{i}=\frac{\sum_{j\;=\;-5}^{5}ARI_{i\;+\;j\;+\;1}-ARI_{i\;+\;j}}{n\sqrt{2}}$$

where *ARI*_*i*_ is the ARI of the *i*th beat and *n* is the number of beats. A schematic representation is shown in Figure 2C.

#### Blood pressure data

Raw BP signals were filtered using a lowpass filter of 40 Hz. Systolic peaks were then located in the filtered BP traces. Systolic BP during the Valsalva was then computed by visually identifying the different phases of the systolic BP signal as described by Palamarchuk et al. (2016). Pressure recovery time (PRT) and systolic overshoot from baseline were calculated allowing assessment of established indices of sympathetic activity (Vogel et al., 2005).

**Figure 3:**
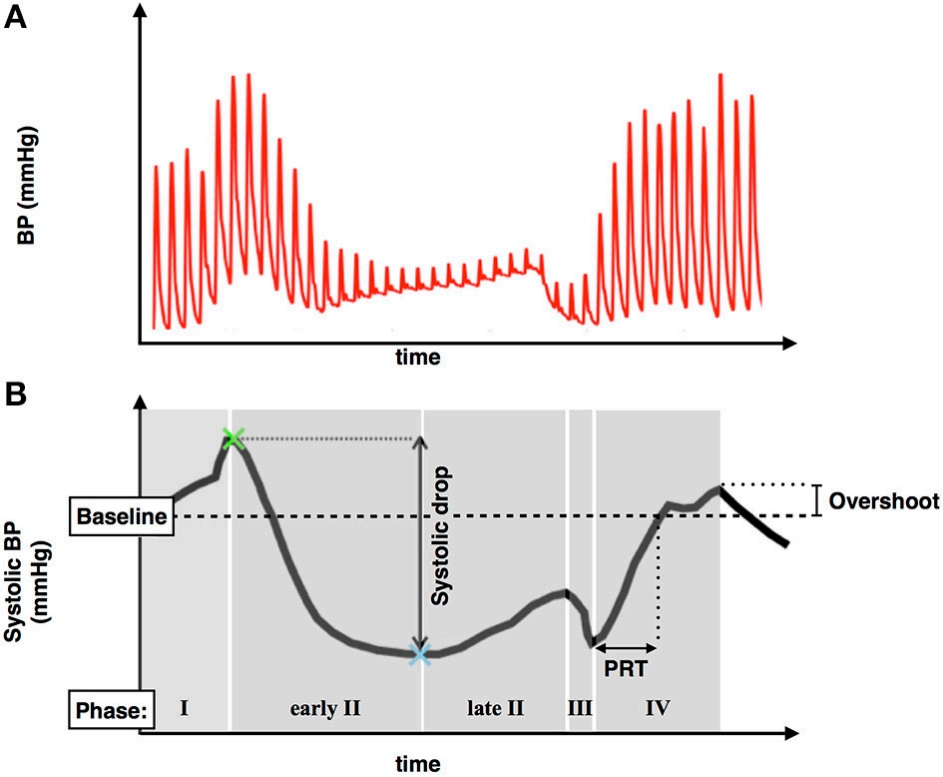
**(A)** Typical blood pressure response during a Valsalva maneuver. **(B)** Systolic blood pressure trace demonstrating phases of the Valsalva, blood pressure drop between phase I and II, pressure recovery time (PRT), and phase IV systolic overshoot.

A typical blood pressure response to the Valsalva maneuver is shown in Figure 3. The subject exhales forcibly against a fixed resistance for 10 s which increases intrathoracic and abdominal pressures thereby impeding venous return, reducing ventricular filling, and reducing left ventricular systolic pressure. An initial increase in blood pressure occurs mainly due to the direct effect of increased intrathoracic pressure (Phase 1). This is followed by a fall in blood pressure due to impeded venous return which reaches a plateau (early phase II) or increases slightly due to reflex sympathetically mediated peripheral vasoconstriction and increased inotropic state of the myocardium (late phase II). Following release of the forced expiration intrathoracic pressure returns to normal and refilling of the pulmonary vascular bed results in a further transient fall in blood pressure (Phase III). As venous return and ventricular filling are restored blood pressure rises and may overshoot due to persisting increased sympathetic tone (Phase IV).

#### Sympathetic nerve activity

Raw SNA signals were bandpass filtered between 500 and 1,000 Hz as in Doytchinova et al. (2017), to remove noise from muscle nerve activity and hence improve signal to noise ratio. In contrast to Doytchinova et al. (2017), patients in this study were paced from the CRT devices. Consequently, much larger pacing artifacts were present in the SNA which were only attenuated (and not fully removed) by the bandpass filtering. More aggressive filtering in the lower range was seen to significantly lower the SNA trace itself, increasing signal-to-noise. Thus, in the cases where residual ECG signal was still present in the signals after filtering, the numerical contribution of the ECG was estimated and removed from the computations involving SNA values. Specifically, this involved not including SNA data within ±20 ms of the pacing spikes.

Changes in SNA during sympathetic stimulation were quantified as in Doytchinova et al. (2017) by computing average values of (filtered) SNA signals occurring within a given temporal window (SNA_av_). Here, SNA_av_ values were computed over a 20 s window during the Valsalva and during a corresponding 20 s period of rest.

### Statistical analysis

For all subjects mean values of Systolic BP, SNA, and STV were averaged across both Valsalva maneuvers. Change following stimulus and comparison between off and on beta-blocker was assessed using two-tailed, paired, *t*-tests. Results were considered significant at *P* ≤ 0.05.

## Results

In the absence of beta-blockade mean baseline systolic blood pressure prior to the Valsalva was 129.6 ± 19.2 mmHg. Mean systolic blood pressures during each phase of the Valsalva were: Phase I = 152.3 ± 23.3 mmHg, Early Phase II = 92.7 ± 25.9 mmHg, Late Phase II = 97.5 ± 25.7 mmHg, Phase III = 65.9 ± 17.3 mmHg, Phase IV 142.8 ± 23.5 mmHg. Analysis of indices of sympathetic function demonstrated a mean overshoot in phase IV from baseline of 13.2 ± 11.1 mmHg and mean PRT of 5.26 ± 4.08 s.

Without beta-blockade all 11 participants demonstrated an increase in mean STV of ARI following the Valsalva. Mean STV increased from 4 ± 1.22 ms at rest to 5.25 ± 0.9 ms (*p* < 0.01). Mean SNA increased from a resting value of 2.3 ± 1–3.7 ± 1.7 uV (*p* < 0.01). Individual blood pressure responses and changes in STV of ARI are presented in Table 2.

**Table 2:** **Individual systolic blood pressure responses and changes in short-term variability (STV) of activation recovery intervals**.

**Subject**	**Bisoprolol**	**Baseline (mmHg)**	**Max phase I (mmHg)**	**Early phase II (mmHg)**	**Late phase II (mmHg)**	**Phase III (mmHg)**	**Phase IV (mmHg)**	**Systolic drop (mmHg)**	**Rise during Phase II (mmHg)**	**Overshoot from baseline (mmHg)**	**PRT (seconds)**	**Rest STV (ms)**	**Valsalva STV (ms)**
1	OFF	114.7	144.8	78.1	85.3	74	128.2	66.7	6.8	13.5	2.99	2.86	6.41
1	ON	130.5	164.4	102.7	101.3	71	135.1	61.7	0	4.6	8.485	4.27	4.89
2	OFF	155.1	181.2	129.1	136	59.3	159.4	52.1	6.9	4.3	5.07	1.58	4.79
2	ON	126.2	139.6	111.7	113.7	78.5	124.5	27.9	2	0	4.895	2.69	4.14
3	OFF	158.4	186.4	125.8	125.1	83.3	175	60.6	0	16.6	2.79	4.04	4.66
4	OFF	137.5	153.3	117.4	120.5	84.5	145.5	35.9	3.1	8	2.715	3.48	3.82
4	ON	112	122.2	101.2	101.5	84.5	120.5	21	0.3	8.5	3.48	2.8	3.84
5	OFF	125.3	132	90.3	103.2	79.1	142.4	41.7	12.9	17.1	2.32	4.5	4.51
5	ON	125.5	131.6	65.2	66.4	48.8	115.9	66.4	1.2	0	6.56	4.7	4.15
6	OFF	132.5	175.1	88.6	88.5	54.5	167	86.5	0	34.5	3.585	4.13	5.97
6	ON	126.3	150.2	75	86.5	67.5	134.5	75.2	11.5	8.2	6.2	4.84	6.01
7	OFF	103.8	123	72	73	44.6	108.1	51	1	4.3	2.45	4.84	5.55
7	ON	96.5	128	97.6	97.3	51.7	103.6	30.4	0	7.1	5.43	4.49	3.76
8	OFF	126	147.5	75.2	87.1	67.5	133.5	72.3	11.9	7.5	6.735	5.62	6.7
8	ON	145.4	174.6	81.2	81.5	61.6	149	93.4	0.3	3.6	7.53	6.05	6.27
9	OFF	100.7	114	42.4	45.4	30.5	100.5	71.6	3	0	13.31	5.23	5.42
10	OFF	148.2	156.9	106.2	110.2	80.4	156.4	50.7	4	8.2	12.86	2.83	4.39
10	ON	166.4	188.9	116.4	119.6	90.6	161.5	72.5	3.2	0	11.835	3.39	3.87
11	OFF	123.5	161.5	94.1	98.3	67.5	154.9	67.4	4.2	31.4	3.045	4.95	5.53

**Figure 4:**
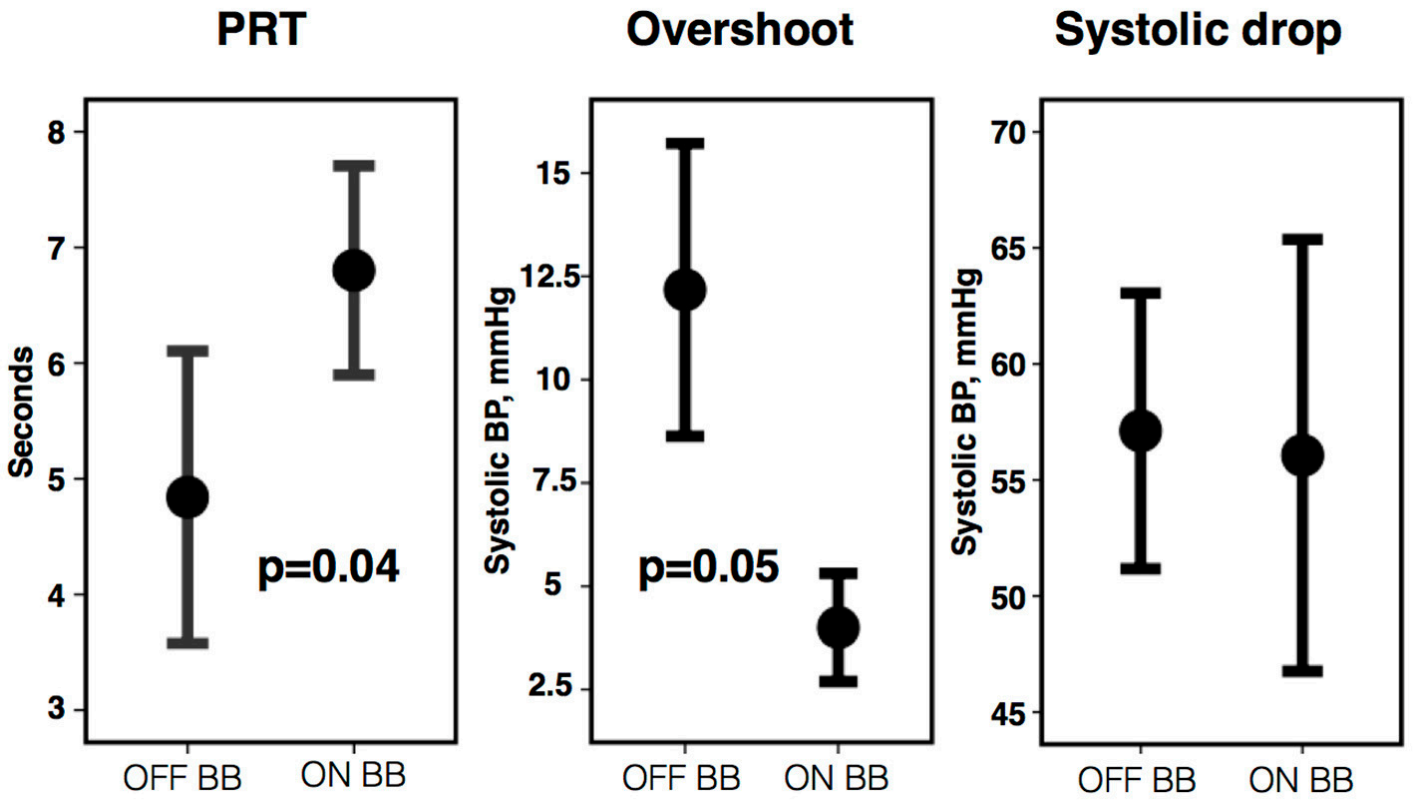
**Pressure recovery time (PRT), phase IV systolic overshoot, and systolic drop from Phase I to II off vs. on bisoprolol (BB)**. Error bars represent ±1 standard error of the mean.

**Figure 5:**
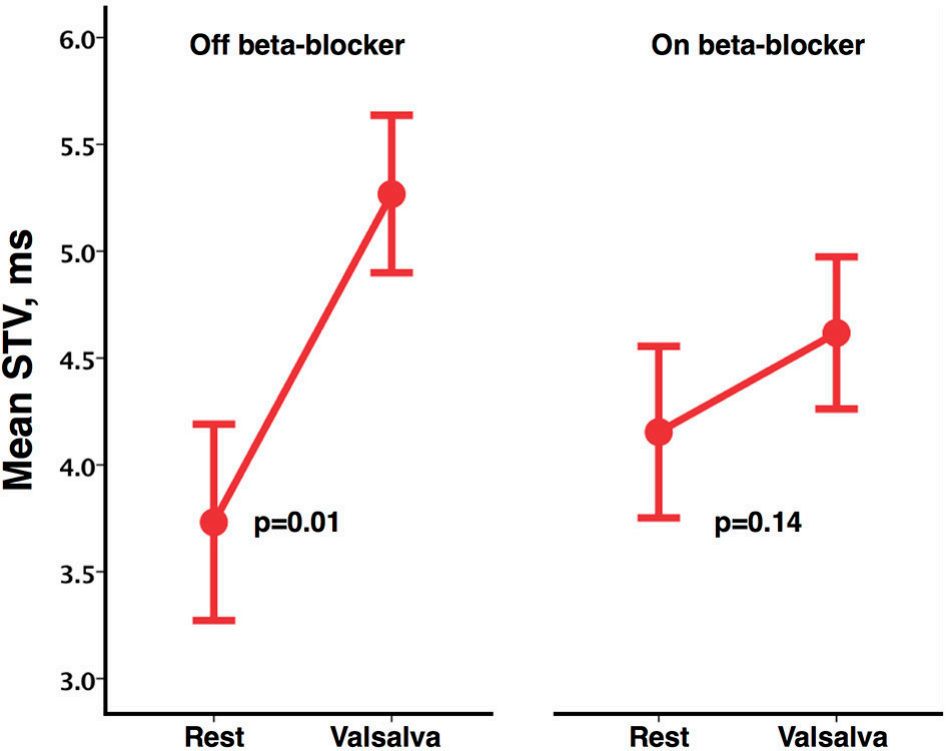
**Mean short-term variability (STV) of activation recovery intervals at rest and following Valsalva, off and on bisoprolol**. Error bars represent ±1 standard error of the mean.

A sub-group of eight participants additionally undertook repeated studies during established beta-blockade (Bisoprolol in all 8). Direct comparison of phases of the blood pressure response and indices of beta-adrenergic function are shown in Table 3. Both beta-adrenergic measures of the Valsalva (systolic overshoot in Phase IV and PRT) demonstrated a statistically significant difference off compared to on bisoprolol (*p* = 0.05 and 0.04 respectively) (Figure 4). This is in keeping with a reduced beta-adrenergic response to the Valsalva when beta-blocked. In the absence of beta-blockade mean STV following the Valsalva increased from 3.73 ± 1.3 ms at rest to 5.27 ± 1.04 ms (*p* = 0.01). When repeated on established bisoprolol mean STV of ARI demonstrated a smaller magnitude of increase from 4.15 ± 1.14 ms at rest to 4.62 ± 1 ms (*p* = 0.14) following the Valsalva, which is also noted to be statistically non-significant. Figure 5 demonstrates changes in STV of ARI off and on bisoprolol. Mean rest SNA off beta-blockers was 2.4 ± 1 uV which increased to 4.4 ± 1.9 uV (*p* = 0.04). When repeated on bisoprolol mean rest SNA was 1.9 ± 0.8 uV and increased to 2.6 ± 0.8 uV (*p* = 0.05).

**Table 3:** **Off and on bisoprolol comparison showing mean systolic blood pressure by phases of the Valsalva, indices of beta-adrenergic function, and changes in short-term variability (STV) of activation recovery intervals**.

	**Baseline (mmHg)**	**Max phase I (mmHg)**	**Early phase II (mmHg)**	**Late phase II (mmHg)**	**Phase III (mmHg)**	**Phase IV (mmHg)**	**Systolic drop (mmHg)**	**Rise during Phase II (mmHg)**	**Overshoot from baseline (mmHg)**	**PRT (seconds)**	**Increase in STV (ms)**
OFF beta-blocker	130.4 ± 16.8	151.7 ± 19.7	94.6 ± 20.9	100.5 ± 20	68 ± 14.1	142.6 ± 19.1	57.1 ± 16.8	5.8 ± 4.7	12.2 ± 10	4.84 ± 3.58	1.54 ± 1.29
ON beta-blocker	128.6 ± 20.8	149.9 ± 24	93.9 ± 18.2	96 ± 17.3	19 ± 15	130.6 ± 18.6	56.1 ± 26.3	2.3 ± 3.9	4 ± 3.7	6.8 ± 2.55	0.46 ± 0.78
*P*-value	0.8	0.86	0.92	0.56	0.82	0.13	0.89	0.22	**0.05**	**0.04**	**0.02**

*Bold values refer to metrics showing statistical significance, i.e., p ≤ 0.05*.

**Figure 6:**
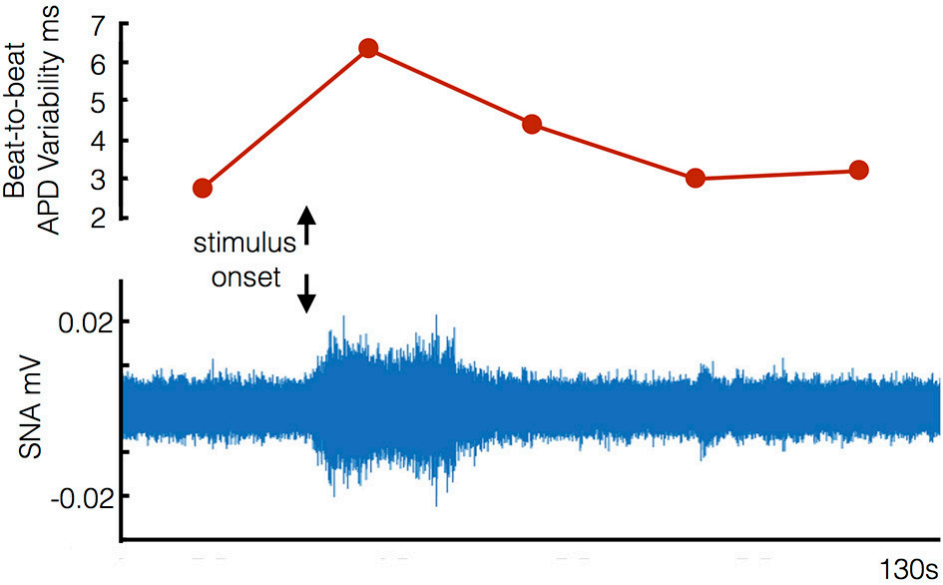
**Example of corresponding increases in beat-to-beat variability of ventricular action potential duration and sympathetic nerve activity (SNA) following stimulus onset (start of Valsalva)**.

Any relative reductions in the magnitude of the changes when on (compared to when off) beta-blockers were specifically analyzed by performing a direct pair-wise comparison of changes from rest during the Valsalva. Following the Valsalva a significantly greater increase in STV of ARI was seen when off beta-blockade (1.54 ± 1.29 ms) compared to when on bisoprolol (0.46 ± 0.78 ms) (*p* = 0.02). Although the observed increases in SNA were higher whilst off beta-blocker (2 ± 1.1 uV) compared to on bisoprolol (0.7 ± 0.4 uV) this was not statistically significant (*p* = 0.07). Figure 6 demonstrates corresponding changes in STV of ARI, and SNA.

## Discussion

Our study employed a novel methodology whereby ventricular APD was measured directly from the epicardium (as ARIs) in ambulatory patients with heart failure during an autonomic challenge induced by the Valsalva maneuver. The main findings were (1) The Valsalva was associated with an increase in BBV-APD; (2) The haemodynamic indices were consistent with increased sympathetic activity as is characteristic of the Valsalva; (3) The Valsalva increased SNA in skin recordings in keeping with a recent validation study (Doytchinova et al., 2017); (4) Beta-blockade with bisoprolol reduced but did not eliminate the increase in BBV-APD.

The Valsalva maneuver is an established method of increasing sympathetic activity whereby forced expiration impedes venous return resulting in a reduction in ventricular pressure and volume, and a baroreflex increase in sympathetic activity (Booth et al., 1962; Korner et al., 1976; Smith et al., 1987). Sympathetic activity has been shown to be greatly enhanced during the strain phase of the Valsalva maneuver in healthy control subjects in studies using microneurographical recordings of muscle sympathetic nerve discharges (Schrezenmaier et al., 2007). Indices of baroreflex sensitivity have been established to separately evaluate the vagal and adrenergic components (Vogel et al., 2005; Schrezenmaier et al., 2007). Patients with severe heart failure may show an altered BP response to the Valsalva with no fall in blood pressure during the strain phase resulting in a “square wave” blood pressure response (Felker et al., 2006). None of our patients exhibited this behavior, all showing a substantial blood pressure drop in phase II (57.1 ± 16.8 mmHg. Mean ± *SD*).

Recent studies have shown that high frequency recordings from skin electrodes reflect stellate ganglion sympathetic nerve activity. These results have been demonstrated in animal models and humans (Jiang et al., 2015; Doytchinova et al., 2017), and have shown that episodes of ventricular tachycardia were preceded by increased skin sympathetic nerve activity within 30 s of onset. Recent studies from Doytchinova et al. (2017) demonstrated an increased skin SNA response to the Valsalva. Whilst further studies are required to elucidate the underlying physiology and utility of skin sympathetic efferent nerve recordings, our results are nevertheless inline with theirs.

To the best of our knowledge this is the first study to demonstrate that BBV-APD is increased during enhanced sympathetic stimulation in humans, and in particular in humans with heart failure. This is important in view of the known association between increased BVR and ventricular arrhythmia and the adverse effects of sympathetic stimulation (Shen and Zipes, 2014; Baumert et al., 2016). Several studies have examined QT interval variability (QTV) in patients with heart failure. Patients with heart failure have a higher QTV compared to age matched controls (Piccirillo et al., 2013). Heart failure patients show a marked circadian variation with an increase in QTV during the daytime (Dobson et al., 2009). Enhanced sympathetic activity by tilt table testing in heart failure patients is associated with an increased QTV although the increase may be impaired in comparison to normal subjects (Desai et al., 2004; Piccirillo et al., 2006). Heart failure patients with a history of ventricular tachycardia (VT) show higher baseline values of QTV compared to those without VT and compared to normal subjects (Nayyar et al., 2013). In the latter study Isoprenaline increased QTV in normal subjects but not in the heart failure-VT group, highlighting the limited autonomic modulation of QTV in heart failure patients (Nayyar et al., 2013).

Beta blockade has shown mixed effects on QTV. For example, no effect at rest (Piccirillo et al., 2001; Nayyar et al., 2013); a reduction during atrial pacing in normal subjects (Mine et al., 2008); no effect on head up tilt increase in QTV in heart failure patients (Nayyar et al., 2013); and reduction of QTV increase during anger recall in post MI patients (Magrí et al., 2012). Circadian variation of QTV is abolished by beta blockade (Furukawa et al., 2006).

The eight patients who were studied on a second occasion whilst on their bisoprolol showed a lessened increase in BBV-APD during sympathetic stimulation compared to when their bisoprolol had been discontinued, although still showing an increase. This could be due to incomplete beta-blockade or to the contribution of an additional mechanism. Such a possibility is mechano-electric feedback in response to the large pressure volume changes during the Valsalva. The effect of mechanical perturbation on APD is complex depending on multiple variables including the type of preparation, the type of stretch e.g., isotonic vs. isometric, and the timing of the stretch in relation to the timing of action potential repolarization. Cellular mechanisms involve stretch activated channels and calcium cycling (Kohl et al., 1998; Quinn and Kohl, 2016). Future studies might address the possibility of a role of mechano-electric feedback in BBV-APD.

Heart rate variability is a major component of QTV. Both APD and the QT interval are strongly cycle length dependent (Boyett and Jewell, 1978; Zaza et al., 1991). Rapid and slow processes are involved with hysteresis effects all of which vary between individuals. Since the majority of QTV studies are conducted in the presence of uncontrolled cycle length, separating rate driven QTV from actual fluctuations in QT interval is technically challenging. Cycle length control by pacing as employed in the present studies avoids these difficulties and removes the component of QTV due to heart rate variability.

A number of factors influence BVR at the cellular level including ion channel stochasticity, APD, restitution properties and calcium handling (Zaniboni et al., 2000; Heijman et al., 2013; Johnson et al., 2013; Baumert et al., 2016; Pueyo et al., 2016). Furthermore, electrophysiological remodeling during heart failure is known to influence a wide range of electrophysiological processes and the molecular mechanisms remain somewhat controversial despite extensive investigation (Nattel et al., 2007; Cho et al., 2012; Kirk and Kass, 2013). At present it is unclear to what extent these factors may interact and be contributory to BVR at the whole heart level, and how it is governed by autonomic stimulus, where electrophysiological changes in individual cells may have a limited effect due to electrotonic coupling in the syncytium. Here, we have shown that sympathetic stimulation directly changes ventricular APDs at the whole heart (coupled tissue) level in the context of heart failure. Additional measurements in future studies of ARI changes in different locations, perhaps with measurements of local strain patterns, as well as changes in conduction velocity may help to further elucidate the driving electrophysiological mechanism at the cellular level.

## Limitations

A limitation of recording from the CRT device is that it is only possible to record from one epicardial electrode while pacing from the right ventricular electrode. Consequently our observations are confined to a single LV site. In view of the well-known regional variation of electrophysiological properties throughout the ventricular myocardium it is possible that other regions may have responded differently. An unavoidable phenomenon of pacing studies is the latency period between the stimulus from the pacing lead and myocardial capture. As a result there is a potential for variability in the cycle length. Analysis of successive activation times demonstrated a maximum variation in heart rate of ±0.3 BPM which was consistent between protocols. This would not be expected to significantly influence our results. An additional unavoidable limitation was the inability to study a control population of normal subjects on account of the necessary requirement of an implanted biventricular pacing device. This would have been of interest on account of the known attenuation of sympathetic responses in heart failure patients. Our study findings are therefore only applicable to patients with heart failure. Finally, this study only involved the use of bisoprolol for beta-blockade. Future investigations may involve assessing how the findings from our study related to other forms of beta-blockade and the dose-response relationship in a larger clinical trial.

## Conclusions

In patients with heart failure (NYHA class I-III) beat-to-beat variability of ventricular action potential duration was increased during an autonomic challenge associated with increased sympathetic activity. These results accord with observations on ECG-QT variability. Our study on ventricular APD in ambulatory heart failure patients provides insight on mechanisms of known importance in the genesis of serious and fatal ventricular arrhythmias.

## Author contributions

BP, MB, JG, and PT conceived and designed the experiments. All authors took responsibility in collecting, analyzing and interpreting the data, with particular individual input in the following areas: electrophysiology (PT), analysis (MB), experimentation (BP). All authors contributed to drafting or revising the manuscript and all authors approved the final version of the manuscript.

### Conflict of interest statement

The authors declare that the research was conducted in the absence of any commercial or financial relationships that could be construed as a potential conflict of interest.
